# Genome Fractionation and Loss of Heterozygosity in Hybrids and Polyploids: Mechanisms, Consequences for Selection, and Link to Gene Function

**DOI:** 10.1093/molbev/msab249

**Published:** 2021-08-19

**Authors:** Karel Janko, Oldřich Bartoš, Jan Kočí, Jan Roslein, Edita Janková Drdová, Jan Kotusz, Jan Eisner, Martin Mokrejš, Eva Štefková-Kašparová

**Affiliations:** 1 Laboratory of Fish Genetics, Institute of Animal Physiology and Genetics of the Czech Academy of Sciences, Liběchov, Czech Republic; 2 Department of Biology and Ecology, Faculty of Science, University of Ostrava, Ostrava, Czech Republic; 3 Department of Zoology, Faculty of Science, Charles University, Prague, Czech Republic; 4 Institute of Experimental Botany, Academy of Sciences of the Czech Republic, Prague, Czech Republic; 5 Museum of Natural History, University of Wroclaw, Wroclaw, Poland; 6 Department of Mathematics, Faculty of Science, University of South Bohemia in České Budějovice, České Budějovice, Czech Republic; 7 IT4Innovations, VŠB—Technical University of Ostrava, Ostrava-Poruba, Czech Republic; 8 Department of Genetics and Breeding, FAFNR, Czech University of Life Sciences Prague, Czech Republic

**Keywords:** hybridization, loss of heterozygosity, gene conversions, hemizygous deletions, polyploidy, asexual reproduction

## Abstract

Hybridization and genome duplication have played crucial roles in the evolution of many animal and plant taxa. The subgenomes of parental species undergo considerable changes in hybrids and polyploids, which often selectively eliminate segments of one subgenome. However, the mechanisms underlying these changes are not well understood, particularly when the hybridization is linked with asexual reproduction that opens up unexpected evolutionary pathways.

To elucidate this problem, we compared published cytogenetic and RNAseq data with exome sequences of asexual diploid and polyploid hybrids between three fish species; *Cobitis elongatoides*, *C. taenia*, and *C. tanaitica*. Clonal genomes remained generally static at chromosome-scale levels but their heterozygosity gradually deteriorated at the level of individual genes owing to allelic deletions and conversions. Interestingly, the impact of both processes varies among animals and genomic regions depending on ploidy level and the properties of affected genes. Namely, polyploids were more tolerant to deletions than diploid asexuals where conversions prevailed, and genomic restructuring events accumulated preferentially in genes characterized by high transcription levels and GC-content, strong purifying selection and specific functions like interacting with intracellular membranes. Although hybrids were phenotypically more similar to *C. taenia*, we found that they preferentially retained *C. elongatoides* alleles. This demonstrates that favored subgenome is not necessarily the transcriptionally dominant one.

This study demonstrated that subgenomes in asexual hybrids and polyploids evolve under a complex interplay of selection and several molecular mechanisms whose efficiency depends on the organism’s ploidy level, as well as functional properties and parental ancestry of the genomic region.

## Introduction

The genome of a typical Metazoan possesses two alleles of each gene brought together by merging reduced gametes of two individuals belonging to the same species. However, these rules have often been alleviated as traces of whole-genome duplications (WGD) and introgressive hybridizations have been documented in many taxa, vertebrates and humans included (Dehal and Boore 2005; Gittelman et al. 2016). Hybridization and polyploidization may cause serious problems, for example, in transcription regulation leading to deregulation of transposable elements (Dion-Côté et al. 2014; Lien et al. 2016) but may also lead to creation of novel traits and acquisition of gene functions via sub-/neofunctionalization of duplicated genes (Yoo et al. 2014; Fridman 2015), potentially facilitating specialization to new niches (Madlung 2013).

Realizing their evolutionary significance and huge practical value to mankind (Mason and Batley 2015), research focused on hybridization and polyploidy intensified and revealed some prominent patterns. For instance, hybrid phenotypes may range from intermediate forms to transgressive expression of novel traits (Bell and Travis 2005; Yoo et al. 2014; Bartoš et al. 2019); however, often, one parental subgenome is more expressed than the other one, known as “expression dominance” (Yoo et al. 2014; Alexander-Webber et al. 2016; Cheng et al. 2018). Hybrid and polyploid genomes evolve dynamically and often lose orthologous genes from one or the other parental species in processes referred to as loss of heterozygosity (LOH), genome fractionation, or rediploidization in polyploids (Yoo et al. 2014; Lien et al. 2016; Du et al. 2020). These processes are often considerably asymmetrical (Alexander-Webber et al. 2016), and it has been proposed that loss of alleles from the less expressed parental subgenome may cause less severe effects and may, therefore, be preferred by selection (Yoo et al. 2014). However, the situation is likely more complex as orthologs may also be lost or retained for proper dosage of molecular interactors and relative copy number of their gene products, that is, selection for stoichiometry (Birchler and Veitia 2012) or due to particular incompatibilities in the interspecific genomic background (Runemark et al. 2018). Hybrid populations may also selectively filter orthologous genes according to their adaptive value in a given environment (Gittelman et al. 2016; Lancaster et al. 2019; Smukowski Heil et al. 2019). Thus, despite the application of modern technologies, the question why some genes tend to be retained in heterozygous or duplicated states, whereas others are subjected to fractionation still represents a major evolutionary puzzle. It remains particularly unclear whether the aforementioned patterns are driven by case-specific mechanisms or whether independent lineages follow similar evolutionary trajectories (Soltis et al. 2010; Deans et al. 2015).

Such a gap in current knowledge partly results from taxonomic bias in knowledge, particularly toward plant species, as the incidence of hybridization and polyploidy have traditionally been underrated by zoologists. Moreover, direct tests for determining adaptive values of genomic rearrangements could be performed only under laboratory conditions, thereby focusing on rapidly reproducing organisms (Smukowski Heil et al. 2017; Lancaster et al. 2019) as events like LOH are rather rare (Dukić et al. 2019). For practical reasons, most available data are derived from natural hybrids and polyploids, making it difficult to discern the patterns that are direct consequences of genome merging and those that evolved subsequently. In addition, many polyploids are of hybrid origin, making it challenging to discern the effects that are inherent to polyploidy and those to hybridization itself. Finally, it is unclear how hybrid and/or allopolyploid taxa could establish themselves in natural environments. This is because any new form is rare at the time of its emergence and is, therefore, threatened by frequency-dependent mating disadvantage and backcrossing with dominating ancestral populations, that is, the minority cytotype exclusion principle (Husband 2000).

New strains may alleviate initial caveats when reproducing asexually, since production of unreduced gametes offers immediate reproductive isolation and clonal multiplication of novel genotypes that is otherwise impossible under sexual reproduction (Cunha et al. 2008; Choleva and Janko 2013; Hojsgaard and Hörandl 2015; Janko et al. 2018; Dubey et al. 2019). The perception of asexual organisms indeed changed among biologists, leading to current appreciation that asexuals occur in all major eukaryotic clades (Schön et al. 2009) and form dominant components in some ecosystems (Kearney 2005; Hojsgaard and Hörandl 2015). The emergence of asexual reproduction is tightly linked to hybridization and polyploidy, reviewed in Choleva and Janko (2013), and may represent an inherent stage of the speciation process, representing a special type of Bateson–Dobzhansky–Muller model (Janko et al. 2018). This paradigm shift coincides with increasing interest in the role of recombination modification in evolution (Thompson and Jiggins 2014; Ortiz-Barrientos et al. 2016). Understanding the evolutionary processes in asexual organisms may thus provide important insights into the mechanisms of genome evolution in general.

Unfortunately, there is no consensus on genomic consequences of asexuality. Clonal inheritance has been originally assumed to ensure stasis of genome with gradual accumulation of deleterious mutations (Muller 1964; Keightley and Otto 2006) and heterozygosity levels (Birky 1996; Mark Welch and Meselson 2000; Balloux et al. 2003) or modified dynamics of transposable elements (Hickey 1982). This view is currently challenged by indications of horizontal gene transfers in some asexual lineages (Gladyshev et al. 2008; Danchin et al. 2010) as well as by accumulating evidence that genomes may acquire aneuploidy or structural changes extremely quickly once the sex is lost, owing to relaxed constraints on the pairing of homologous chromosomes (Triantaphyllou 1981; Sunnucks et al. 1996; Normark 1999; Spence and Blackman 2000; Tucker et al. 2013). Heterozygosity may degrade quickly by hemizygous deletions and particularly by gene conversions (Tucker et al. 2013), which may lead to increased GC content in asexual genomes (Bast et al. 2018). Notably, the dynamics of asexual genome may also be determined by its mode of origin; nonhybrid asexuals appear to have lost most of their heterozygosity as a consequence of pervasive gene conversions, whereas hybrid asexuals generally express high levels of heterozygosity, probably indicating efficient clonal transmission of the parental genomes (Jaron et al. 2021).

**Fig. 1.**
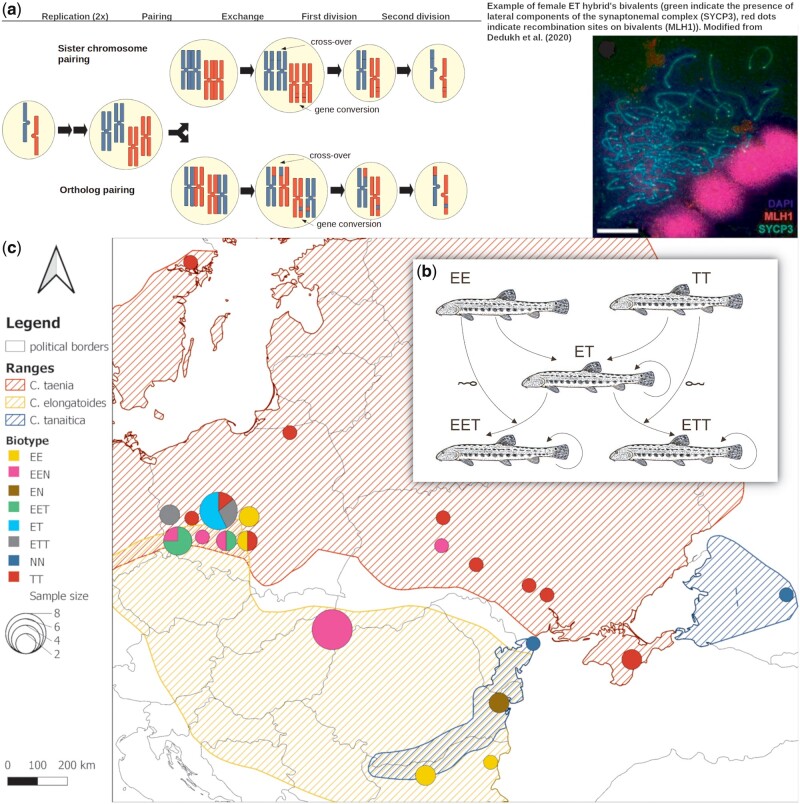
The *Cobitis taenia* hybrid complex. (*a*) Example of gametogenetic pathways (adapted from Lutes et al. [2010]) involving endoreplication and followed by meiotic pairing of either sister chromosomes (upper pathway) or orthologous chromosomes (lower pathway). Note that the latter case may cause loss of heterozygosity among progeny via crossovers or gene conversions. Inset on the right demonstrates empirical evidence for the presence of proper bivalents in hybrid’s oocytes as from Dedukh et al. (2020). (*b*) Reproduction scheme of *Cobitis*; letters correspond to haploid genomes: E = *C. elongatoides*, N = *C. tanaitica*, T = *C. taenia*. (*c*) Map of species distribution and samples’ origin; sites’ numeric code corresponds to supplementary table S1, Supplementary Material online.

With such varying patterns, it is difficult to discern the mechanisms that are taxon-specific and those that are related to asexual reproduction per se. A major complication is that the so-called asexual organisms form a very heterogeneous group by employing a wide spectrum of gametogenetic mechanisms, ranging from processes with very distorted meiosis (apomixis) to automictic pathways employing more or less normal meiotic divisions (Stenberg and Saura 2009; Stenberg and Saura 2013). Some types of automixis involve intragenomic recombinations or exclusions of large genomic parts, thereby decreasing heterozygosity among the progeny (Bi and Bogart 2006), whereas other types have genetic consequences equivalent to mitosis. For example, many hybrid asexuals employ “premeiotic endoduplication,” a mechanism wherein normal meiosis is preceded by WGD in oogonial cells. As a consequence, segregation and recombination presumably occur on bivalents between sister copies of the chromosomes rather than between the orthologs, resulting in clonal progeny (Lutes et al. 2010; Arai and Fujimoto 2013) (fig. 1*a*).

In this study, we analyzed the causes and consequences of allelic recombination, conversion, and LOH in a clonally reproducing vertebrate of hybrid origin, *Cobitis* (Actinopterygii). We focused on the so-called *Cobitis taenia* hybrid complex, which arose by hybridization of the species *C. elongatoides* (we have denoted its haploid genome as “E”) with either of its two distant relatives, *C. taenia* (denoted as “T”) or *C. tanaitica* (denoted as “N”) (Choleva et al. 2012). Phylogenomic analysis (Janko et al. 2018) revealed that *C. taenia* diverged from *C. tanaitica* relatively recently, approximately 1 Ma (million years ago) but the initial E–(TN) divergence occurred approximately 9 Ma and was initially followed by intensive gene exchange. However, with ongoing divergence, these species lost the capacity to produce sexual hybrids as crossings of the current species led to sterile hybrid males but clonally reproducing, fertile hybrid females. Hybrid females form unreduced gametes by premeiotic endoduplication but are gynogenetic, that is, they require sperm from a sexual species to trigger development of their gametes (Janko et al. 2007; Choleva et al. 2012; Dedukh et al. 2020). Usually, the sperm genome is degraded after fertilization, but a certain proportion of oocytes fuse with sperm cells, and consequently, diploid ET or EN females produce a certain portion of triploid progeny that may have EET, ETT, EEN, or ENN genomic constitution, depending on the sperm donor. Triploids are also gynogenetic and reproduce clonally (fig. 1*b*).

Hybridization between the parental species is reciprocal, but *C. taenia* is the maternal ancestor of most *elongatoides*–*taenia* hybrids, whereas *C. elongatoides* is the maternal ancestor of *elongatoides*–*tanaitica* hybrids (Janko et al. 2003). Because parental species lack the obvious prezygotic reproductive barriers and glacial–interglacial cycles repeatedly brought their ranges into contact (fig. 1*c*), new clones arose dynamically throughout much of the Pleistocene epoch and subsequently colonized Europe (Janko et al. 2005, 2012). Current asexual populations therefore consist of recent *elongatoides–taenia* clones with postglacial origin in the Central European hybrid zone, as well as of ancient *elongatoides*–*tanaitica* hybrids (the so-called hybrid clade I consisting of EN and EEN biotypes), which originated in the Balkan hybrid zone approximately 300 ka (kilo years ago) (Janko et al. 2005).

*Cobitis* hybrids, thus, offer excellent opportunity to investigate how genomes of natural asexual organisms evolve through time and across different ploidy levels. Majtánová et al. (2016) demonstrated by karyotypic analysis that clonal hybrids maintain remarkable integrity of the parental chromosomes without traces of large-scale recombinations and restructuring, despite more than 300 ky of evolution since the initial hybridization event. In this study, we investigated the dynamics of diploid and polyploid clonal genomes on the finer scale of individual genes. To achieve this aim, we performed exome sequencing of the sexual parental species and their clonal hybrids and subsequently compared the data with recently published gene expression profiles (Bartoš et al. 2019). This allowed us to identify mechanisms underlying fractionation and LOH in clonal genomes and to test how they relate to expression and function of the affected genes.

## Results

### Polymorphism Detection and Identification of Species-Specific Variants

**Fig. 2.**
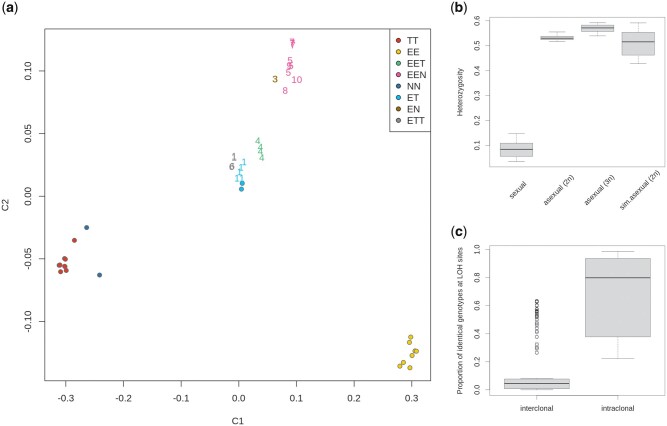
(*a*) Multidimensional scaling (MDS; SVD algorithm) of individual samples based on filtered SNPs; clustering visualization of first two coordinates (Plink v1.9b) validates samples’ genetic origin; letters in the legend correspond to haplotype genomes as follows: E* *=* C. elongatoides*, N* *=* C. tanaitica*, T* *=* C. taenia*. Hybrid individuals are denoted by numerals, which indicate the ID of determined clonal lineages (MLLs), as in supplementary table S2, Supplementary Material online (note that clone-mates tend to be clustered in the MDS plot). (*b*) Heterozygosity of sexual and asexual (split to diploids and triploids) samples estimated across all filtered variant sites; missing sites are not included. Expected heterozygosity levels for diploid asexuals are depicted on last boxplot as obtained by simulation from parental individuals. (*c*) Boxplots indicate the proportions of shared genotypes at LOH positions among all pairs of individuals belonging to the same (right) and different (left) clonal lineages. Note that we used the set of E–TN diagnostic sites to maintain compatibility of *elongatoides*–*taenia and elongatoides*–*tanaitica* hybrids.

Exome-capture data were acquired from 46 specimens, including three parental species and their asexual hybrids sampled across all ploidy types and hybrid genome compositions (see fig. 1*c* and supplementary table S1, Supplementary Material online, for details). We also included whole-genome sequences of one ET hybrid for control. In addition, we took special care to minimize the problem of missing some single nucleotide polymorphism (SNP) variants of parental species and therefore selected parental individuals from previously defined regions. As such, we covered all major phylogroups and zoogeographical provinces of their ranges as defined by Janko et al. (2005). Reads were mapped against published *C. taenia* reference transcriptome containing 20,600 contigs (Janko et al. 2018), and for simplicity we restricted our analysis to 189,927 quality-filtered biallelic SNPs (i.e., SNPs occurring in no more than two states across the entire data set). The greatest genetic divergence was between *C. elongatoides* and the remaining two sister species, *C. taenia and C. tanaitica*, whereas hybrids appeared intermediate (multidimensional scaling [MDS]; fig. 2*a*). Examination of pairwise genetic distances revealed that hybrid individuals cluster into 11 groups with nonrandomly high similarity among individuals within clusters and low similarity among clusters. In accordance with Arnaud-Haond et al. (2007) and our previous work (Janko et al. 2012), we refer to such clusters as multilocus lineages (MLL) and consider them as representatives of independent clonal lineages descending from distinct hybrid origin or, in the case of triploids, distinct polyploidization events (see fig. 2*a* and supplementary table S2, Supplementary Material online).

To characterize hybrid’s SNP variation relative to their parental species, we followed Ament-Velásquez et al. (2016) and divided all hybrids’ SNPs into ten categories (supplementary table S2, Supplementary Material online), of which five were particularly important for this study. First, we identified the so-called “private-asexual” SNPs (i.e., categories pr1a and pr1b in the supplementary table S2, Supplementary Material online), where hybrids possessed unique variant not occurring among parental species. These SNPs presumably represent mutations acquired after clonal origin (Ament-Velásquez et al. 2016; Kočí et al. 2020) and we detected 16,372 such unique positions in total. The proportions of such SNPs in genome of each hybrid were notably correlated with its mtDNA distance from the nearest sexual counterpart (Pearson’s *r* = 0.971, df = 23, *P*-value = 8.66e-16), so that the ancient *elongatoides*–*tanaitica* hybrids had the highest number of private asexual SNPs, whereas experimental F1 hybrids had the lowest number of such SNPs, probably representing only rare sequencing errors.

Second, we focused on SNP variants that were diagnostic between pairs of parental species, so that their hybrids should possess one or both parental variants, thereby allowing detection of LOH events. Throughout the entire data set, we therefore identified sites diagnosing *C. elongatoides* from *C. taenia* (referred to as E–T diagnostic sites; total of 37,988), *C. elongatoides* from *C. tanaitica* (E–N diagnostic sites; total of 30,281), and we also found SNPs differentiating *C. elongatoides* from the joint data set of *C. taenia and C. tanaitica* (E–TN diagnostic sites; total of 27,311). According to the way how individual hybrids’ SNPs were shared with these parental variants, we categorized them as “shared SNPs” of type sh3a (heterozygous for both parental variants), sh3b11 (homozygous for one parent’s allele), and sh3b12 (homozygous for the other parent’s allele). Their numbers per each hybrid individual are provided in supplementary table S2, Supplementary Material online.

In addition, the supplementary table S2, Supplementary Material online, also contains other possible states, such as heterozygous sites private to one sexual species, that are either shared (prh2a) or unshared with asexual hybrids (prh2b and prh2b1) and heterozygous sites shared by both sexual species that also do (sh4a) or do not (sh4b) appear heterozygous in the asexual hybrid.

### Clonal Lineages Accumulate Loss of Heterozygosity Events in Their Evolution

Hybrids were considerably more heterozygous than parental species (fig. 2*b*; Wilcoxon rank sum test: *W* = 520, *P*-value < 1e-9) with no less than 98.5% of private asexual SNPs and the vast majority of diagnostic sites occurring in heterozygous states. The levels of heterozygosity in diploid hybrids were lower than among triploids but achieved values expected by combining the individuals of extant parental species (fig. 2*b*; last boxplot). Nevertheless, LOH was observed in some portion of diagnostic SNPs of every hybrid (categories sh3b11 and sh3b12 in supplementary table S2, Supplementary Material online). We verified the quality of base-calling and LOH detection by two approaches. We first compared SNP calling from exome-capture technology and whole-genome sequencing of the same ET hybrid (csc067) and found differences in only approximately 0.17% of E–T diagnostic positions. We also compared two F1 hybrids against their parents and found homozygous states only in approximately 0.3% of positions, where both parental individuals differed from each other. As these variants were suspiciously present in most of the other specimens, suggesting potential sequencing or demultiplexing errors rather than real variants, we excluded them from subsequent analyses. Overall, this indicates high reliability of LOH detection based on exome capture.

**Fig. 3.**
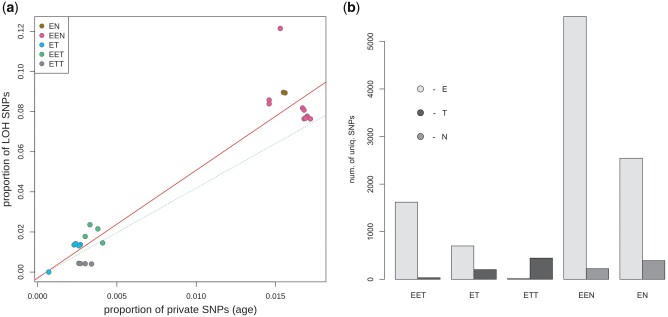
(*a*) Correlation between proportion of private hybrid SNPs as the proxy for asexual individual’s age (*x*-axis), and proportion of LOH loci (*y*-axis) in each individual. For plotting points and their linear regression (solid line) we used E–T diagnostic sites for *elongatoides*–*taenia* hybrids and E–N diagnostic sites for *elongatoides*–*tanaitica* hybrids. Dashed line represents the linear regression fit for data calculated on E–TN diagnostic sites used for all individuals (points not shown). Letters correspond to haplotype genomes as follows: E = *C. elongatoides*, N = *C. tanaitica*, T = *C. taenia*. (*b*) Barplot showing proportions of LOH events according to their genomic origin. Height of each bar represents absolute number of unique SNPs that appeared as LOH in a given biotype (note that barplots are not corrected for number of individuals pooled in respective biotypes).

Two patterns were noted in the distribution of LOH SNPs. First, individual LOH sites were significantly more likely to be shared by individuals belonging to the same clonal lineage (MLL) than by individuals from different clones (Wilcoxon rank sum test, *W* = 4,540, *P*-value < 1e-9; fig. 2*c*). Second, the proportion of LOH sites in each individual significantly correlated with its proportion of private asexual mutations (Pearson’s *r* = 0.955, 95% CI = 0.902–0.980, *P*-value = 3.286e-14; fig. 3*a*, supplementary table S2, Supplementary Material online). Hence, although we may not rule out existence of somatic mutations (López and Palumbi 2019), our data indicate that erosion of heterozygosity is heritable within clonal lineages, accumulates over clone’s evolutionary history, and therefore affects the germline.

We are aware that some sites may gain an apparent LOH state due to ancestral polymorphism, when same allele might have been inherited from both parents at time of clonal origin but was subsequently lost in one parental species. To minimize this type of error, we also analyzed the E–TN diagnostic sites, most of which presumably became diagnostic long before the origin of studied clones. This is because *C. taenia—C. tanaitica* divergence predates origin of the oldest *Cobitis* clones by hundreds of thousands of years (Janko et al. 2018). Yet, proportions of LOH at E–TN diagnostic sites also correlated significantly with the private asexual SNPs (*r* = 0.948, 95% CI = 0.885–0.977, *P*-value = 2.145e-13) albeit with slightly less steep slope than in ET– and E–N sites, respectively (fig. 3*a*). This suggests that some false positives might have affected our data set, but altogether the retention of ancestral polymorphism is an unlikely explanation of most observed LOH events.

### Heterozygosity Deteriorates by Gene Conversions and Hemizygous Deletions in Asexuals

We next investigated topological context of LOH sites to test whether they might have been generated by point mutations. Since we used cDNA reference that excludes introns, we could not simply analyze the physical distance between SNPs in studies loci. Instead, we tested whether LOH sites within individual genes tend to occur in contiguous stretches; to do so, we compared the scores characterizing the contiguity of observed LOH sites with permuted data sets with LOH sites randomly distributed across all genes (see the Materials and Methods for details). We found that empirical values calculated from real data sets exceeded the highest simulated value of any permutated data set, suggesting that most LOH sites tend to occur in clusters and LOH events are created by processes like gene conversions and deletions that affect contiguous stretches of DNA.

**Fig. 4.**
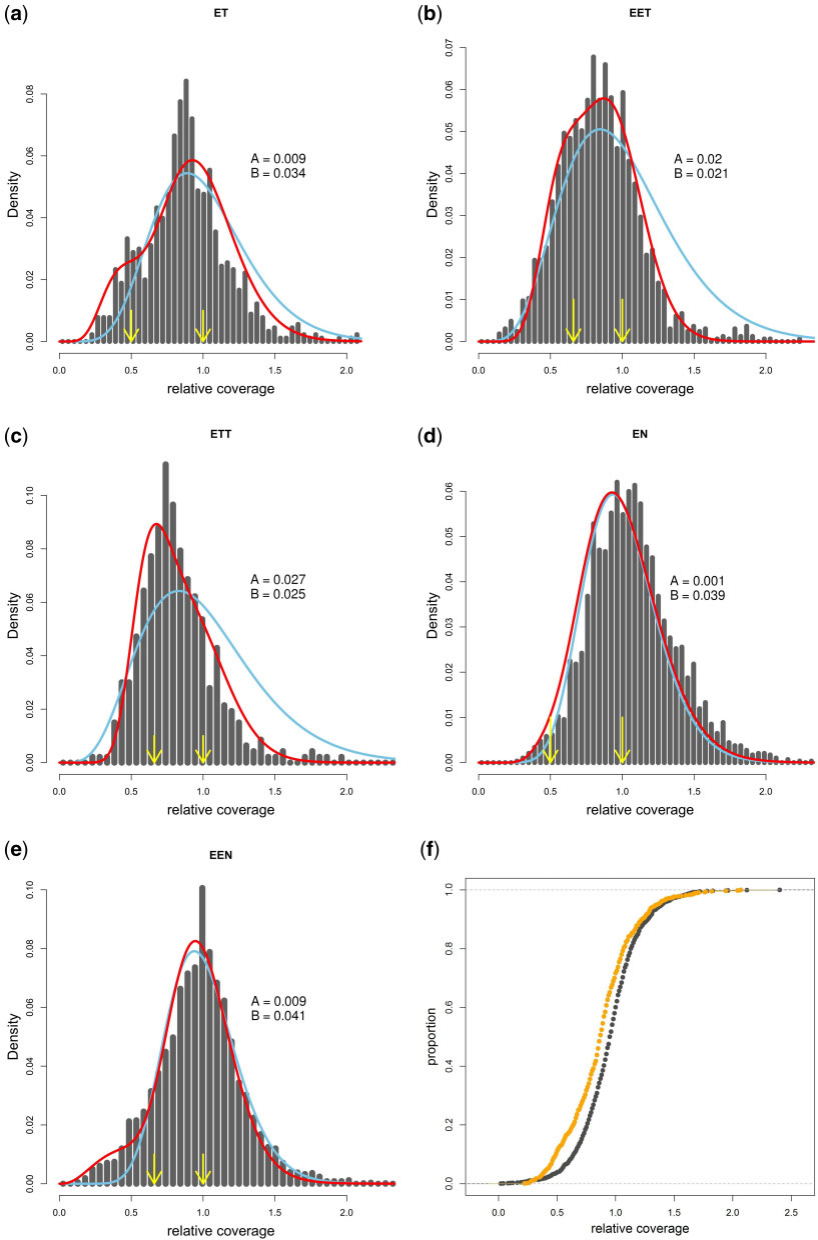
(*a*–*e*) Histograms of relative coverages at LOH loci in ET, EET, ETT, EN, and EEN hybrids pooled into respective biotypes. Arrows depict biologically meaningful values (for given ploidy); blue lines represent the fit of single Gamma distribution with mean centered at value 1, red lines represent the fitted mixture of two Gamma distribution with the coefficients A and B indicating proportions of both Gamma distributions in the combined model; A relates to the distribution assuming the mean relative coverage approximately 1, B to the distribution with the mean approximately 0.5 or 0.66 (for diploid or triploid biotype, respectively); (*f*) Orange represents empirical cumulative distribution function (ECDF) of relative coverages at LOH sites of ET biotype, black represents ECDF of relative coverages at the same sites, but taken from parental species, where no deletions are expected. ECDF curves for all biotypes and results of KS test are provided in supplementary figure S1, Supplementary Material online.

To distinguish between both candidate processes, we analyzed the sequencing coverage following Tucker et al. (2013), who showed that conversions conserve the amount of allelic copies, whereas allelic deletions would result in coverage drop. Given that targeted sequencing may provide considerable variance in coverage across loci, we first tested whether our exome capture data are suitable for coverage comparisons among individuals and across loci like in other studies (Duvaux et al. 2015; Bragg et al. 2016) (see supplementary appendix S1, Supplementary Material online). We than used normalized per-SNP coverages to calculate relative values of coverage for each hybrids’ LOH by comparing it with the coverages of the same site in parental species (see Materials and Methods). Following Tucker et al. (2013) we predicted that conversions result in relative coverage approximately 1, whereas allelic deletions would result in coverage drop to values approximately 0.5 in diploids, or approximately 0.66 (single deletion) and approximately 0.33 (double deletion) in triploids. To investigate roles of both processes in LOH creation, we constructed for each hybrid biotype the histograms of relative coverages and tested their modality at aforementioned biologically relevant values. Ancient clones (EN and EEN) possessed relatively smooth distributions of relative normalized coverages with peaks close to 1, suggesting gene conversions as the main mechanism causing their LOH events. In contrast, recent clones (ET, EET, and ETT) had additional peaks located at lower values (fig. 4*a*–*e*), indicating simultaneous operation of both processes (Tucker et al. 2013).

To formally test whether observed data may be explained by single process or several simultaneously operating processes, we compared the fits of each histogram by single gamma distribution as a proxy for operation of only one process, or mixtures of more distributions with means fixed at aforementioned biologically relevant values. Nonlinear least square method was used to estimate the parameters controlling relative proportions of distributions in combined models (A and B parameters control the rate of conversions vs. hemizygous deletions in two-gamma distribution model, whereas A, B, and C control the rates of conversions vs. hemizygous vs. double deletions in three-distribution model).

The most complex model assuming the occurrence of conversions and both single and double deletions (three gamma distributions) did not significantly improve the fit to triploids’ data. However, mix of two gamma distributions assuming gene conversions and hemizygous deletions significantly outperformed any single-distribution in most data sets (*F*-test in diploids: EN: df = {40,38}, *F* = 1.44 (critic.val. = 1.71); ET: df = {41,39}, *F* = 7.87 (critic.val. = 1.69); triploids: EEN: df = {44,42}, *F* = 11.01 (critic.val. = 1.66); EET: df = {44,42}, *F* = 112.6 (critic.val. = 1.66); ETT: df = {31,29}, *F* = 57.49 (critic.val. = 1.84)). This suggests that both processes operate jointly in all hybrids with the possible exception of EN diploids, where the null hypothesis that its LOH has accumulated due to gene conversions only could not be rejected. In theory, if the true mean of hybrid’s normalized coverages is below 1, the mixed-distribution model may be preferred even if the distribution of coverage fits a single gamma model. We thus fitted a fourth model assuming single gamma distribution with free mean. We compared its fit to the preferred mixed-gamma model byAkaike Information Criterion adjusted for small sample size (AICc) calculated from least squares, because such models are not nested. We found that mixed two-gamma model outperformed the single gamma model with free mean in two out of five hybrid biotypes (EET: ΔAICc = 3.07; EEN: ΔAICc = 5.29), corroborating the hypothesis of simultaneous occurrence of conversions and deletions in *Cobitis* hybrids.

To further validate whether deletions indeed occur in hybrids, we used Kolmogorov–Smirnov test to compare whether distributions of relative coverages at hybrids’ LOH sites significantly differ from relative coverages at exactly same sites in parental species, where no deletions are expected. These differences proved significant in all biotypes (see fig. 4*f* and supplementary fig. S1, Supplementary Material online, for details). The ecdf curves indicated that all hybrid biotypes have an excess of low-coverage LOH sites, again suggesting that deletions exist in hybrids.

Both tests thus documented the existence of LOH sites with decreased DNA content suggesting that LOH events are generally caused by simultaneous operation of conversions and hemizygous deletions in *Cobitis* hybrids. However, double deletions in triploids appear very rare, albeit their existence is indicated by retention of haploid allele in some LOH sites.

### Accumulation of LOH Is Biased with Respect to Parental Subgenome, Ploidy, and Hybrid Type

We noted that LOHs were nonrandomly distributed among hybrids, and there were several trends behind such unevenness. First, there was a clear bias in retention of parental subgenomes (fig. 3*b*). In triploids, vast majority of detected LOH sites possessed allele of that parent which contributed two chromosomal sets. This type of bias in triploids is likely methodological, since losses of one allele of the diploid subgenome would still appear heterozygous and hence escape our attention. However, significant bias was observed in diploid hybrids with preferential retention of *C. elongatoides* allele at approximately 80% LOH sites in ET and at approximately 87% in EN hybrids.

Second, the hemizygous deletions were significantly more common in triploid hybrids than in their diploid counterparts. Specifically, the A/(A + B) ratio of combined Gama distributions suggests that deletions accounted for only approximately 21% LOH events in ET diploid hybrids, whereas their contribution rose to approximately 50% in triploid EET and ETT hybrid forms (fig. 4*a*–*c*). Similarly, in *elongatoides–tanaitica* hybrids, the mixed gamma model indicated that deletions accounted for less than 0.1% of LOH events in diploid EN hybrids (and in fact, it did not significantly outperform the conversion-only model), whereas triploid EEN possessed approximately 18% of deletions at LOH sites (fig. 4*d and e*). We tested the significance of these differences by comparing the fit to diploid data sets of a “free” mixed gamma model with A/B ratios optimized to ET, and of “forced” models where the A/B ratios were fixed to values estimated from corresponding triploids (EET). Specifically, the values for the “forced” models were taken from the 95% CI of the A/B ratio provided by the fitting algorithm in triploids, and we found that such “forced” models provided significantly worse fits than free model applied to the ET data set (df = {40,39}, *F* = 14.97, critical value = 1.7); EN versus EEN data sets were not tested as the mixed gamma model did not outperform the single gamma in EN, as it did in triploids.

Finally, hemizygous deletions appeared significantly more common among recent asexuals than in ancient clones, as evident from comparisons of A/B ratios between recent (ET) and ancient (EN) diploid clones (∼21% vs. ∼0.1%) as well as of recent (EET or ETT) and ancient (EEN) triploid clones (∼50% vs. ∼18%) (fig. 4*a*–*e*) (EEN fitted with 95% CI values of A/B taken from EET data: df = {43,42}, F = 78.4 (critic.val. = 1.67)).

### Occurrence of LOH Is Related to Sequence Composition, Allelic Expression, and Gene Function

#### LOH Depends on GC Content but Patterns Are Complex

**Fig. 5.**
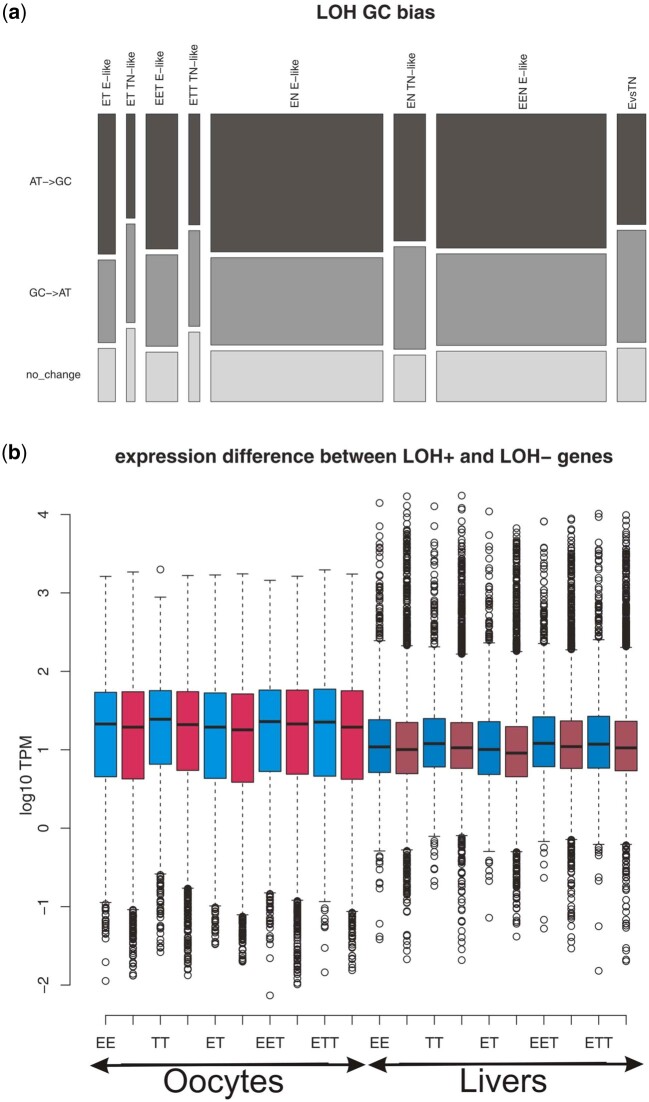
(*a*) G/C bias at asexuals’ LOH sites as demonstrated for one representative of every biotype. Each individual is represented by two columns with E-like (left) and T-like (right) LOH events. Bar widths scale with absolute numbers of observed LOH events in each individual, whereas heights of color fields demonstrate proportions of LOH causing weak to strong, strong to weak, and no GC change. The last bar (E vs. TN) represents overall differences between C. *elongatoides* and both other parental species at all E–TN diagnostic sites. Note the consistent increase in weak to strong substitution rates in E-like LOH events of all hybrids as compared with interparental divergence. The application of contingency tables indicated significance of this trend in all cases, except the F1 hybrids and one ET specimen (csc052). By contrast, no such shift has been detected at TN-like LOH sites. (*b*) TPM normalized expression characteristics of LOH positive (blue) and LOH negative (red) genes in all *Cobitis* biotypes analyzed by Bartoš et al. (2019) (individuals are pooled into biotypes). For better orientation, the oocyte data (left part) are depicted in lighter color tones, whereas liver data (right part) are in darker tones.

To investigate potential GC bias, we first inspected transcriptome-wide GC contents of sexual and asexual forms, measured either across all positions or only at the relatively neutral third-codon positions, but found no significant differences between any biotypes. Next, we performed a more fine-scale analysis on E–TN diagnostic positions and separated all detected LOH sites into E-like or TN-like groups, depending on parental allele retained. Comparing parental sequences with each hybrid we inferred how many LOH sites underwent A/T→G/C substitutions, G/C→A/T substitutions, or no change in GC content (i.e., A↔T or G↔C substitutions). We used contingency tables to compare these counts with overall A/T—G/C differences between respective parental species across all E–TN diagnostic positions and found that E-like LOH events were significantly biased in favor of A/T→G/C substitutions in triploid (EET, EEN) and diploid (ET, EN) biotypes. This bias was approximately 21% on average and proved significant after false discovery rate (FDR) correction in every individual, except for both F1 hybrids and one ET hybrid (csc052). In contrast, we observed no significant GC bias in TN-like LOH sites of any biotype (fig. 5*a*). Our data thus indicate that GC-dependence has complex background and occurs only during loss of *taenia*/*tanaitica* allele, but not in the opposite direction.

#### LOH Is Affected by Gene’s Transcription

We evaluated the effects of allele expression on LOH occurrence using recently published transcription profiles of livers and oocytes in sexual (*C. elongatoides*, *C. taenia*) and asexual (ET, EET, and ETT) females (Bartoš et al. 2019). Although Bartoš et al. analyzed different individuals, they used the same reference transcriptome and thus we could investigate the transcription profiles of those genes which carried E–T diagnostic SNPs and were either LOH-positive (E-like or T-like LOH bearing), or LOH-negative, according to present exome-capture results. We applied two approaches to test for differences in gene and allele expression between these categories.

First, we found that LOH events tend to occur in genes with above-average expression levels. Specifically, we compared the TPM-normalized (transcript per million) expression levels of Bartoš et al.’s (2019) data and found that the genes where present analysis discovered LOH events had significantly higher expression levels in liver tissue of all biotypes (FDR corrected WMW test *P*-values < 0.02 for all biotypes). Same trends, albeit insignificant, were observed in oocytes.

Second, we used allele-specific expression data normalized by the total-count approach from Bartoš et al. (2019) to test whether less expressed alleles tend to be preferentially lost during LOH events. Specifically, we applied WMW test with FDR correction to compare the distributions of *C. elongatoides*/*C. taenia* allelic log_2_ fold change in hybrids (Ehyb/Thyb log2FC) from Bartoš et al. (2019) between genes where present study discovered LOH event versus genes where no LOH has been found.

In a result, we found that genes with E-like LOH events tend to have higher Ehyb/Thyb log2FC than genes where no LOH was detected. Such difference proved significant after FDR correction at following data sets: oocytes in ET hybrids (mean log2FC at genes with Elike LOH 0.156 vs. mean log2FC at genes without LOH −0.022); oocytes in EET hybrid (0.0889 vs. 0.042); livers in EET hybrid (0.0965 vs. 0.038); oocytes in ETT hybrids (0.1076858 vs. −0.2557641); livers in ETT hybrids (0.0542 vs. −0.2566). This indicates that less expressed allele (T-allele) is usually lost at E-like LOH genes. By contrast, no significant differences were found on T-like LOH genes with the exception of ETT biotype, where the Ehyb/Thyb log2FC was also significantly higher than at non-LOH genes (oocytes in ETT hybrids: −0.01199159 vs. −0.25576413; livers in ETT hybrids: 0.02149824 vs. −0.25667614). Hence, at least in ETT, the more expressed allele (E-allele in this case) seems to be preferentially lost during T-like LOH events.

It appears, therefore, that the fate of hybrid’s alleles is affected by its expression levels but the effect is again not symmetrical with respect to subgenome ancestry.

#### LOH Accumulates in Genes with Specific Functions

Finally, we investigated whether LOH events accumulate in genes with specific functions. For this purpose, we performed two tests on loci with diagnostic SNPs that were successfully annotated. First, as a proxy for selection regime of particular genes, we used *d*_N_/*d*_S_ values among orthologous sequences of *C. elongatoides*, *C. taenia*, and *C. tanaitica* published by Kočí et al. (2020) and found in both *elongatoides*–*taenia and elongatoides*–*tanaitica* hybrid types that LOH-positive genes were characterized by significantly lower *d*_N_/*d*_S_ values than LOH-negative genes (linear mixed effect model with pairs of individuals as random factor; LRT *P*-value for *elongatoides*–*taenia* = 1.604095e-135; *P*-value for *elongatoides*–*tanaitica* = 1.518951e-05).

Next, we searched whether LOH-positive genes are associated with particular Gene Ontology (GO) terms using the GO::TermFinder (Boyle et al. 2004). Resulting lists of top 20 enriched GO terms of each category in each biotype are provided in table 1 (cellular compartment GO terms) and supplementary table S3, Supplementary Material online (biological process and molecular function GO terms). Results show that GO terms associated with membrane coats and endoplasmatic reticulum were enriched among LOH-positive genes in EET, EN, and EEN biotypes with *P*-values corrected for multiple tests below alpha level 0.1. It also shows that some GO terms, whose corrected *P*-value exceeded the threshold level, were repeatedly encountered among top enriched GO terms in several hybrid biotypes including independently arisen *elongatoides*–*taenia and elongatoides*–*tanaitica* hybrid types. These namely contained cellular compartment type GO terms associated with cell–cell junction and cell–substrate adherence junction and biologic processes type GO terms of cellular biogenic amine metabolic process and protein glycosylation**.**

**Table 1:** Top 20 Enriched GO Terms in Cellular Compartment GO Category Ranked by Their *P-*value in Each Hybrid Biotype.

GO ID	GO Description	*P*-value and Numbers of LOH-Positive Genes versus Total Number of Genes
ET (292 LOH positive genes, 4,582 annotated with diagnostic SNP)		
GO:0005576	Extracellular region	pV 0.0028351 (22 vs. 184)
GO:0005923	Bicellular tight junction	pV 0.0161340 (3 vs. 9)
GO:0070160	Tight junction	pV 0.0219788 (3 vs. 10)
GO:0000347	THO complex	pV 0.0222870 (2 vs. 4)
GO:0000445	THO complex part of transcription export complex	pV 0.0222870 (2 vs. 4)
GO:0016021	Integral component of membrane	pV 0.0338391 (76 vs. 987)
GO:0031224	Intrinsic component of membrane	pV 0.0353113 (76 vs. 989)
GO:0031680	G-protein beta/gamma-subunit complex	pV 0.0355939 (2 vs. 5)
GO:0097431	Mitotic spindle pole	pV 0.0355939 (2 vs. 5)
GO:0005834	Heterotrimeric G-protein complex	pV 0.0366592 (3 vs. 12)
GO:0043296	Apical junction complex	pV 0.0366592 (3 vs. 12)
GO:1905360	GTPase complex	pV 0.0366592 (3 vs. 12)
GO:0030687	Preribosome, large subunit precursor	pV 0.0552198 (3 vs. 14)
GO:0000346	Transcription export complex	pV 0.0686844 (2 vs. 7)
GO:0000152	Nuclear ubiquitin ligase complex	pV 0.0878187 (2 vs. 8)
GO:0005911	Cell–cell junction	pV 0.0891895 (4 vs. 27)
GO:0000922	Spindle pole	pV 0.0897273 (3 vs. 17)
GO:0031234	Extrinsic component of cytoplasmic side of plasma membrane	pV 0.0897273 (3 vs. 17)
GO:0043230	Extracellular organelle	pV 0.1083001 (2 vs. 9)
GO:0070062	Extracellular exosome	pV 0.1083001 (2 vs. 9)
ETT (226 LOH positive genes, 4,582 annotated with diagnostic SNP)		
GO:0005844	Polysome	pV 0.0287870 (3 vs. 14)
GO:0031514	Motile cilium	pV 0.0318521 (2 vs. 6)
GO:0005730	Nucleolus	pV 0.0390230 (11 vs. 123)
GO:0005732	Small nucleolar ribonucleoprotein complex	pV 0.0431628 (2 vs. 7)
GO:0071007	U2-type catalytic step 2 spliceosome	pV 0.0431628 (2 vs. 7)
GO:0030684	Preribosome	pV 0.0446155 (6 vs. 53)
GO:0044798	Nuclear transcription factor complex	pV 0.0577484 (4 vs. 30)
GO:0090575	RNA polymerase II transcription factor complex	pV 0.0577484 (4 vs. 30)
GO:0031974	Membrane-enclosed lumen	pV 0.0808439 (31 vs. 488)
GO:0043233	Organelle lumen	pV 0.0808439 (31 vs. 488)
GO:0070013	Intracellular organelle lumen	pV 0.0808439 (31 vs. 488)
GO:0005657	Replication fork	pV 0.0839463 (2 vs. 10)
GO:0034708	Methyltransferase complex	pV 0.0990699 (4 vs. 36)
GO:0071013	Catalytic step 2 spliceosome	pV 0.1015022 (3 vs. 23)
GO:0005793	Endoplasmic reticulum–Golgi intermediate compartment	pV 0.1228584 (3 vs. 25)
GO:0031461	Cullin-RING ubiquitin ligase complex	pV 0.1456830 (3 vs. 27)
GO:0032040	Small-subunit processome	pV 0.1456830 (3 vs. 27)
GO:0071944	Cell periphery	pV 0.1530158 (23 vs. 373)
GO:0035097	Histone methyltransferase complex	pV 0.1697646 (3 vs. 29)
GO:0031981	Nuclear lumen	pV 0.1815560 (23 vs. 382)
EET (662 LOH positive genes, 4,582 annotated with diagnostic SNP)		
GO:0030117	Membrane coat	pV 0.0007544 (12 vs. 31)
GO:0048475	Coated membrane	pV 0.0007544 (12 vs. 31)
GO:0030120	Vesicle coat	pV 0.0014970 (9 vs. 21)
GO:0030662	Coated vesicle membrane	pV 0.0137591 (9 vs. 28)
GO:0031224	Intrinsic component of membrane	pV 0.0145280 (165 vs. 989)
GO:0016021	Integral component of membrane	pV 0.0172777 (164 vs. 987)
GO:0030126	COPI vesicle coat	pV 0.0186441 (4 vs. 8)
GO:0031314	Extrinsic component of mitochondrial inner membrane	pV 0.0186441 (4 vs. 8)
GO:0016020	Membrane	pV 0.0189391 (234 vs. 1456)
GO:0005921	Gap junction	pV 0.0208470 (2 vs. 2)
GO:0030057	Desmosome	pV 0.0208470 (2 vs. 2)
GO:0042627	Chylomicron	pV 0.0208470 (2 vs. 2)
GO:0071782	Endoplasmic reticulum tubular network	pV 0.0208470 (2 vs. 2)
GO:0098827	Endoplasmic reticulum subcompartment	pV 0.0208470 (2 vs. 2)
GO:0030663	COPI-coated vesicle membrane	pV 0.0297850 (4 vs. 9)
GO:0005911	Cell–cell junction	pV 0.0322002 (8 vs. 27)
GO:0012506	Vesicle membrane	pV 0.0322119 (11 vs. 42)
GO:0030659	Cytoplasmic vesicle membrane	pV 0.0322119 (11 vs. 42)
GO:0030660	Golgi-associated vesicle membrane	pV 0.0395767 (8 vs. 28)
GO:0030687	Preribosome, large subunit precursor	pV 0.0402965 (5 vs. 14)
EN (755 LOH positive genes, 3,197 annotated with diagnostic SNP)		
GO:0005789	Endoplasmic reticulum membrane	pV 0.0006197 (46 vs. 126)
GO:0072546	ER membrane protein complex	pV 0.0007271 (5 vs. 5)
GO:0042175	Nuclear outer membrane–endoplasmic reticulum membrane network	pV 0.0009193 (46 vs. 128)
GO:0031090	Organelle membrane	pV 0.0041040 (107 vs. 364)
GO:0012505	Endomembrane system	pV 0.0071884 (116 vs. 405)
GO:0005783	Endoplasmic reticulum	pV 0.0075373 (53 vs. 166)
GO:0005924	Cell–substrate adherens junction	pV 0.0098916 (5 vs. 7)
GO:0005925	Focal adhesion	pV 0.0098916 (5 vs. 7)
GO:0030055	Cell–substrate junction	pV 0.0098916 (5 vs. 7)
GO:0099081	Supramolecular polymer	pV 0.0121988 (19 vs. 49)
GO:0099512	Supramolecular fiber	pV 0.0121988 (19 vs. 49)
GO:0016528	Sarcoplasm	pV 0.0131308 (3 vs. 3)
GO:0016529	Sarcoplasmic reticulum	pV 0.0131308 (3 vs. 3)
GO:0017087	Mitochondrial processing peptidase complex	pV 0.0131308 (3 vs. 3)
GO:0042383	Sarcolemma	pV 0.0131308 (3 vs. 3)
GO:0030176	Integral component of endoplasmic reticulum membrane	pV 0.0134881 (12 vs. 27)
GO:0031227	Intrinsic component of endoplasmic reticulum membrane	pV 0.0134881 (12 vs. 27)
GO:0030016	Myofibril	pV 0.0147223 (6 vs. 10)
GO:0030017	Sarcomere	pV 0.0147223 (6 vs. 10)
GO:0005813	Centrosome	pV 0.0251815 (12 vs. 29)
EEN (1,235 LOH positive genes, 3,197 annotated with diagnostic SNP)		
GO:0012505	Endomembrane system	pV 0.00017207 (190 vs. 405)
GO:0005783	Endoplasmic reticulum	pV 0.0008575 (84 vs. 166)
GO:0042175	Nuclear outer membrane–endoplasmic reticulum membrane network	pV 0.0009040 (67 vs. 128)
GO:0005789	Endoplasmic reticulum membrane	pV 0.0009619 (66 vs. 126)
GO:0072546	ER membrane protein complex	pV 0.0007271 (5 vs. 5)
GO:0030176	Integral component of endoplasmic reticulum membrane	pV 0.0087823 (17 vs. 27)
GO:0031227	Intrinsic component of endoplasmic reticulum membrane	pV 0.0087823 (17 vs. 27)
GO:0030496	Midbody	pV 0.0095585 (8 vs. 10)
GO:0005794	Golgi apparatus	pV 0.0153109 (69 vs. 145)
GO:0005924	Cell–substrate adherens junction	pV 0.0154665 (6 vs. 7)
GO:0005925	Focal adhesion	pV 0.0154665 (6 vs. 7)
GO:0030055	Cell–substrate junction	pV 0.0154665 (6 vs. 7)
GO:0045121	Membrane raft	pV 0.0201926 (7 vs. 9)
GO:0098589	Membrane region	pV 0.0201926 (7 vs. 9)
GO:0098857	Membrane microdomain	pV 0.0201926 (7 vs. 9)
GO:0097525	Spliceosomal snRNP complex	pV 0.0259453 (10 vs. 15)
GO:0031090	Organelle membrane	pV 0.0351521 (157 vs. 364)
GO:0030532	Small nuclear ribonucleoprotein complex	pV 0.0456027 (10 vs. 16)
GO:0031300	Intrinsic component of organelle membrane	pV 0.0534485 (28 vs. 56)
GO:0031301	Integral component of organelle membrane	pV 0.0534485 (28 vs. 56)

Note.—For each hybrid biotype, we indicate number of genes affected by LOH event and total number of annotated genes with diagnostic SNP relevant for given combination of parental species. For each GO term, we indicate its ID, description and uncorrected *P*-value, as well as numbers of LOH-positive genes and total number of genes with given GO in parentheses. Underlined GO terms are significant after correction for multiple tests at alpha level = 0.1. Background colors are used to highlight GO terms shared between distinct biotypes so that the same color across biotypes indicates GO terms that are identical or nested. In particular, orange indicates genes related to intrinsic component of membrane; gray the genes related to cell–cell junction; dark yellow the genes related to endoplasmic reticulum; dark green the genes related to endomembrane system; and light green the genes related to organelle membrane.

## Discussion

Genomes of asexual organisms may evolve in various ways, ranging from fast restructuring to long-term conservation of heterozygosity. Such diversity of patterns may reflect the variety of gametogenetic pathways used by such organisms and supposedly, hybrids employing premeiotic endoreplication, such as *Cobitis*, can maintain integrity of the parental subgenomes without any chromosomal-scale restructuring (Majtánová et al. 2016). However, the present study showed that despite apparent stasis on a large scale, the heterozygosity gained during original hybridization may gradually deteriorate by small-scale restructuring that affects genomic regions in relation to allelic origin, sequence composition, and gene expression.

### Large-Scale Stasis versus Small-Scale Dynamics of Asexual Genomes

After initial merging and duplication, allopolyploid organisms appear to deduplicate their genomes prominently via fractionation and deletions of orthologs (Yoo et al. 2014; Cheng et al. 2018; Du et al. 2020). However, we found that deletions accounted for a rather minor fraction of genomic restructuring events in polyploid loaches and especially in diploid hybrids. A majority of LOH sites had relative coverage close to 1, thereby indicating higher incidences of recombination between orthologs. Recombination may be followed by crossover (CO), which is expected to cause long stretches of LOH spanning till another recombination site or until the telomeric ends of the paired chromosomes (fig. 1*a*). However, as the cytogenetic study by Majtánová et al. (2016) ruled out any large-scale exchange of chromosomal arms between subgenomes, it appears that most LOH events detected in this study were caused by gene conversions without COs.

Interestingly, it is unclear how such conversions between orthologs may arise since organisms employing premeiotic endoreplication should rather form bivalents between sister copies of the duplicated homologs (Lutes et al. 2010; Arai and Fujimoto 2013; Dedukh et al. 2020) (fig. 1*a*). In theory, they may result from errors in homology search during early meiosis if ExT bivalents are formed, but this explanation is unlikely for two reasons. First, *C. elongatoides and C. taenia* karyotypes are so divergent that most orthologous chromosomes may not form proper bivalents, leading to sterility of hybrid forms that lack endoreplication, typically males (Dedukh et al. 2020). Hence, even if ectopic ExT pairings occur in cells of asexual females, the formation of proper bivalents would be unlikely and gametes would not be formed. Second, Dedukh et al. (2020) documented the occurrence of true COs in ExE and TxT bivalents in hybrid females (fig. 1*a*) suggesting that hypothetical ExT bivalents would result in the exchange of large pieces of chromosomal arms, which was not observed (Majtánová et al. 2016). An alternative explanation would, therefore, assume the role of mitotic conversions, which are important in DNA damage repair (Helleday 2003). Indeed, mitotic conversions have been hypothesized to impact the evolution of asexuals (Omilian et al. 2006; Mandegar and Otto 2007), although, to the best of our knowledge, they have not yet been directly observed in any multicellular asexual organism. It is also possible that genes affected by conversions are localized to specific regions, particularly prone to interchromosomal concerted evolution (Polanco et al. 1998), but such questions may not be answered without precise chromosome-level whole-genome assembly that is currently unavailable.

Whatever the underlying mechanism, the fact that LOH sites are heritable and shared among clone-mates suggests that LOH events occur in the germline. The genes affected by LOH events also clearly possess some characteristics typical of loci undergoing conversions. Namely, LOH-positive genes have above-average expression levels, which is consistent with the hypothesis that DNA of transcriptionally active loci is more relaxed and, hence, prone to double-strand breaks (DSB), followed by repair cascade, including the recombination machinery (González-Barrera et al. 2002; Cummings et al. 2007). *Cobitis* hybrids also tend to replace the less expressed parental allele by the more expressed allele, which is in-line with growing evidence that more transcribed homoeologs are preferably utilized as templates during DSB-induced gene conversion (Schildkraut et al. 2006). Finally, the prevalence of AT→GC substitutions on some LOH sites conforms to the expected GC bias in template preference (Duret and Galtier 2009; Williams et al. 2015).

Interestingly though, our results indicate that processes affecting asexual genomes may depend on the ancestry of the allele acting as a template. Namely, both the preferential retention of the more transcribed allele and the AT→GC substitution bias were apparent only during *taenia→elongatoides* allele replacement (E-like LOHs). In fact, we found some indication that different mechanisms occur during the opposite direction (T-like LOHs) as the more expressed allele tends to be lost in ETT hybrids. However, overall GC contents were not notably affected by these processes as we found no differences between sexual and asexual forms at the transcriptome-wide scale. This suggests that the predicted increase in GC content (Bast et al. 2018) cannot be generally applied to all types of asexuals and the ancestry of subgenomes should be taken into account.

### Impact of LOH on Evolution of Hybrids and Polyploids

Genome rearrangements may bring both, the benefits as well as the constraints to the asexual organism, and their accumulation may be facilitated by the lack of requirement of proper homology for chromosomal pairing (Sunnucks et al. 1996). Consequently, conversions and deletions of genes or even chromosomal arms may potentially proceed at faster rates than mutation accumulation in some asexuals (Triantaphyllou 1981; Sunnucks et al. 1996; Normark 1999; Spence and Blackman 2000; Tucker et al. 2013). Hence, they may slow mutational deterioration (e.g., Muller’s ratchet process) by erasing deleterious mutations or increasing the fixation rate of beneficial mutations (Khakhlova and Bock 2006; Mandegar and Otto 2007). However, recombination per se may have mutagenic effects on its own (Arbeithuber et al. 2015).

In any case, recent analysis of mutation accumulation and fitness deterioration proposed several reasons why LOH events do not play important role in slowing the Muller’s ratchet in asexual loaches (Kočí et al. 2020). In brief, approximately only <1.5% of private asexual SNPs occur in homozygous states, indicating a rather efficient mechanism of clonal reproduction, when a majority of newly acquired mutations occur in heterozygous states on one chromosome with little possibility of recombination or conversion. Consequently, the observed rate of LOH accumulation was low occurring in only approximately 8% of investigated interspecific SNPs after approximately 300 ka of evolution in the oldest clone. This is orders of magnitude less than that in aforementioned taxa, wherein such processes have been hypothesized to interfere with the accumulation of deleterious mutations. Finally, the efficiency of LOH in erasing mutations should increase with clonal age. Initially, the rarely occurring LOH events in recent clones would likely happen on genes without any accumulated mutations. In contrast, in older clones with many more deleterious mutations accumulated throughout their genomes, any LOH event has a higher chance to affect previously mutated genes in older clones. Such an age-dependent process is expected to produce exponential rather than linear correlation between the proportions of LOH and private asexual SNPs. However, this expectation was not met by Kočí et al.’s (2020) data. This is not to say that LOH events may not counteract the ratchet in other asexuals; however, we suggest that their role in removal of deleterious mutation is supposedly smaller in organisms with relatively efficient clonal reproduction, such as *Cobitis*.

Nevertheless, we found that LOH events may considerably impact the evolution of the studied asexuals by other mechanisms, as will be discussed in following paragraphs.

#### Effects of Deletions Are Less Severe in Polyploids

Hemizygous deletions cause aneuploidies on subchromosomal levels and may, therefore, modify the stoichiometry between interacting components of molecular complexes (Birchler and Veitia 2012) or between transcription factors and their binding sites, thereby affecting gene regulation (Veitia et al. 2013). The magnitude of their effect probably scales with the length of the deleted genomic region, that is, long deletions affecting many genes have stronger effect than short-range deletions (Veitia et al. 2013). This may explain why *Cobitis* hybrids retained stable karyotypes with no chromosomal-scale deletions (Majtánová et al. 2016), but small-scale deletions of individual genes do occur and are not removed by selection. Veitia et al. (2013) further postulated that the impact of aneuploidy should depend on the allelic dosage. Consequently, hemizygous deletions would have weaker effects in triploids (changing allelic copy numbers from 1 to 2/3, which is relative to the rest of genome) than those in diploids (changing from 1 to 1/2), and double deletions in triploids (change changing from 1 to 1/3) would have the most severe effects. This may explain why triploids possessed higher proportion of hemizygous deletions than their diploid counterparts, but in the same time, indications of double deletions were rarely observed.

The hypothesis that allelic deletions have a mostly negative impact may also explain why young clones possess a relatively higher proportion of deletions than old clones. Indeed, selection-based removal of deleterious mutations requires some time proportional to the selection coefficient, population size, and genetic background, and hence, although recent clones acquired lower absolute numbers of LOH events, they would have a higher fraction of deletions due to a time-lag necessary to remove these deleterious mutations (Johnson and Howard 2007). Similar differences between young and old clones were reported by Kočí et al. (2020) with regard to relative loads of nonsynonymous mutations, suggesting that young clones may accumulate deleterious mutations, like deletions, in higher rates because the ratchet has not yet reduced their fitness to a critical level or because fewer mutations produce less potentially costly epistatic interactions.

#### Biased Genome Fractionation and Template Preference

Another prominent pattern was the strong preference for *elongatoides* subgenome retention at LOH sites (fig. 3*b*). Biased genome fractionation is commonly observed among hybrids and allopolyploids and may have various explanations, ranging from mechanistic reasons, when one ortholog induces the other’s loss, to natural selection, preferring the fixation of one allelic type in hybrid populations. For instance, fractionation bias has been put in context to subgenome expression dominance in hybrids (Yoo et al. 2014; Alexander-Webber et al. 2016; Cheng et al. 2018). Mechanisms causing such expression dominance are unclear and may relate to various processes such as cis-/trans-divergence, unequal content of transposable elements, or levels of heterochromatinization among parental species (Woodhouse et al. 2014; Bottani et al. 2018). In any case, it has been proposed that once expression dominance occurs, loss of homoeologs from the lower-expressed subgenome would be preferred by selection due to less severe consequences (Yoo et al. 2014).

Interestingly, our data contrast with this prediction, as the preferentially retained subgenome—*elongatoides*—was clearly not dominant in hybrids. Instead, Bartoš et al.’s (2019) data showed significant bias toward *taenia*-like expression of ecologic and phenotypic traits and overall expression level dominance of the *taenia* subgenome in hybrid transcriptomes with slight total prevalence of *taenia* transcripts in somatic tissue (∼1.5%) and germline (∼4%) of diploid hybrids. This suggests that expression dominance is not the causal explanation for biased genome fractionation in *Cobitis* hybrids.

Selective elimination of one parental subgenome may be particularly adaptive in gynogens by increasing their similarity to the parental species that provides them with the sperm, thereby increasing the chance to be fertilized (Beukeboom and Vrijenhoek 1998). Interestingly, all investigated ET hybrids coexist with *C. taenia*, making it unlikely that the preferential loss of *C. taenia* alleles provides such type of sex-mimicry.

Our data, thus, suggest that the causal link between transcriptome-wide expression dominance and biased genome fractionation is more complex than that predicted by the aforementioned hypotheses. For instance, Bartoš et al. (2019) documented that magnitude of *C. taenia* expression dominance differs between somatic traits and germline, suggesting that biased genome fractionation may reflect tissue-specific expression characteristics and other traits that often escape a researcher’s attention.

We may also speculate that biased retention of the *C. elongatoides* subgenome may reflect its special “mechanistic” properties. Several reasons for biased template preference have already been proposed, including different expression levels of orthologous alleles (Schildkraut et al. 2006), different GC contents (Duret and Galtier 2009; Williams et al. 2015), or the effect of maternal ancestry when maternal endonuclease systems may preferentially induce DSB on paternal chromosomes, thereby causing biased DSB repair and unequal gene conversion (Wang et al. 2010). However, none of these hypotheses may sufficiently explain the prevalence of E-like LOH as the *C. elongatoides* subgenome possesses neither higher expression levels nor different GC content and the maternal ancestor of all studied *elongatoides*–*taenia* hybrids was *C. taenia*. The observed bias may thus reflect other phenomena, such as specific distribution of epigenetic markings and methylation, which are known to affect recombination landscape (Mirouze et al. 2012), and may acquire unexpected and nonadditive patterns in hybrids compared with their parents (Hegarty et al. 2011).

Nevertheless, although we did not identify the proximate reason for biased subgenome retention, our data do indicate that both subgenomes differ in their likelihood and mechanism to induce LOH events in hybrids. For instance, we observed a higher proportion of LOH events in EET triploids than those in ETT triploids (fig. 3). Given that detection of LOH events is limited in triploids by the presence of two conspecific allelic copies, a higher fraction of allelic loss/replacements would escape our attention in ETT than in EET triploids if E-like LOH occurs more frequently than T-like LOH. Moreover, we already mentioned that E-like and T(N)-like LOH events differ with respect to allelic expression or GC bias, suggesting some fundamental differences between hybrid subgenomes in the likelihood and mechanisms to induce allelic loss or replacement.

### LOH Preferentially Accumulates in Particular Gene Pathways

Benefits of LOH in hybrids has been directly tested in only a few studies (Smukowski Heil et al. 2017, 2019). Even in these cases, the benefits of LOH appeared quite complex and specific for a given allele, subgenome, and environmental conditions (Lancaster et al. 2019). Although we could not directly examine the effects of LOH, our study revealed some parallel trends in LOH among independent hybrid strains. This suggests that LOH tends to accumulate in genes characterized by some common expression and functional properties, such as higher-than-average expression levels and lower-than-average *d*_N_/*d*_S_ ratios, indicating strong purifying selection to maintain their functionality.

Furthermore, we found that some gene pathways appear to be more affected by accumulation of LOH events than others, as apparent from the significant enrichment of GO terms associated with endomembrane systems, endoplasmic reticulum, and coated vesicles in some biotypes (table 1). We have no explanation for such observation at the moment, and in theory, the enrichment of certain GO classes may just reflect aforementioned expression characteristics of LOH-positive genes. However, it is worth mentioning that detected GO terms often involve genes participating in multimeric protein complexes that ensure vesicle tethering, coating, and transport to membranes. As subunits of such complexes are coevolving to maintain proper functionality, it is tempting to speculate that hybrids would profit from removal of heterozygosity because a mix of protein interactors from diverged orthologs may negatively impact composition of the entire complex.

Unfortunately, the power of GO analysis was weakened by the relatively low number of annotated genes with diagnostic SNPs in a manner that some GOs appeared insignificant after *P*-value correction, albeit all their genes carried LOH in some biotypes (table 1). Interestingly, same or nested GO terms were sometimes recorded among top-enriched GO terms of different hybrid biotypes, thereby hypothetically indicating LOH accumulation also in other gene pathways, which would, therefore, be worth studying further, for example, the concerned GO terms associated with cell–cell junction; cell–substrate adherence junction, containing genes expressed since the early zygotic embryogenesis that take part in cell migration; and cell–cell communication (Siddiqui et al. 2010; Goonesinghe et al. 2012; Matsui et al. 2015).

## Conclusions

There is increasing evidence suggesting that genomes of asexual, hybrid, and polyploid taxa evolve dynamically with selective filtering of parts of the parental subgenomes. Some common trends have been identified in genome evolution of unrelated hybrid/polyploid taxa, for example, in relation to gene/allelic expression. Although patterns revealed in the present study were generally consistent with several previously reported trends, our data do not support some widely cited ideas, such as preferential retention of transcriptionally dominant subgenome. The study on asexual hybrid loaches, thus, revealed that genome fractionation is a very complex process involving simultaneously operating mechanisms that range from a priori bias in template selection to selective fixation of adaptive LOH variants. Our study demonstrated that the relative impact of involved mechanisms likely depends on the reproductive mode, origin of particular subgenome, allelic sequence composition and transcription activity as well as on properties of involved genes and environmental conditions. In combination with recent advances in understanding the effect of aneuploidies (Birchler and Veitia 2012; Veitia et al. 2013), the data acquired on taxa, such as asexual hybrid loaches, can provide invaluable insight into the role of gene dosage in genome evolution in hybrids and neopolyploids. Investigation of genome evolution in hybrid and polyploid taxa may also provide important information about fundamental biological processes, such as meiosis and mitosis.

## Materials and Methods

### Studied Specimens

The study is based on exome-capture data from 46 specimens including *C. paludica* as outgroup, three parental species *C. elongatoides*, *C. tanaitica, C. taenia* and their asexual hybrids of various genomic compositions (see fig. 1 and supplementary table S1, Supplementary Material online, for details). The specimens were a priori categorized into taxonomical units using flow cytometry and published PCR-RFLP markers (Janko et al. 2007). As in Janko et al. (2018), we also included two laboratory *elongatoides–taenia* F1 hybrids with their parental individuals as a control of quality of base calling and LOH site detection.

### DNA Sequencing, SNP Calling, and Identification of Clonal Lineages

Isolated gDNA was sheared with Bioruptor to proper fragment distribution, tagged by indices, pooled, hybridized to exome-capture probes designed by Nimblegen Company based on custom-provided reference transcriptome (Janko et al. 2018). Probes were designed as 80mers with considerable overlap to minimize SNP-specific probe dropouts. Captured fragments were sequenced on Illumina NextSeq in 75-bp paired-end mode. To verify the robustness of exome-capture data and subsequent interpretation, we also performed whole-genome shotgun sequencing of single ET hybrid (csc067) using the HiSeq X Ten sequencing platform in paired-end mode (average fragment length 200 bp, library preparation and sequencing performed by Macrogen). Obtained reads were quality-trimmed by fqtrim tool (Pertea 2015); minimum read length 20 bp; 3′-end trimming if quality drops below 15 and aligned to *C. taenia* reference transcriptome that was published by Janko et al. (2018) who employed several measures to minimize the occurrence of potentially paralogous contigs. In brief, they used the least heterozygous species of Cobitidae (*C. taenia*) for reference assembly, and after mapping reads from different species, they excluded all contigs with spurious heterozygosity, potentially indicative of (pseudo-)paralogy. To identify mitochondrial variants, we also mapped the reads to published *C. elongatoides* mitochondrion (accession number: NC_023947.1). Mapping was performed with BWA MEM algorithm (Li and Durbin 2009) and resulting files were processed with Picard tools (http://broadinstitute.github.io/picard/). Individuals’ variants were called with GATK v3.4 HaplotypeCaller tool and all individuals were jointly genotyped using the GenotypeGVCFs tool (McKenna et al. 2010; DePristo et al. 2011; Van der Auwera et al. 2013). Variant quality score recalibration was based on available database of species-diagnostic positions (Janko et al. 2018) representing learning set for variant quality score recalibration tool VariantRecalibrator. This tool uses machine learning on a data set of known reliable variants to subsequently recalibrate quality scores of target variants in whole-sequence capture data set. Recalibrated variants were then filtered with ApplyRecalibration tool using 90% tranche to filter all variants. All resulting highly confident SNPs with coverage ≥10 and genotype quality ≥20 were transferred into the relational database using our own Python3/SQL scripts.

SNP data of each specimen were subjected to clustering analysis by Plink v1.90b4 (Chang et al. 2015). To simplify the analysis, we focused solely on biallelic SNPs with at most two variants throughout the entire data set. This resulted in removal of approximately 1‰ of positions. We also removed 103 positions where the two laboratory F1 hybrids differed from their parents, because such variants were suspiciously present in most of the other specimens and suggested potential sequencing or demultiplexing errors rather than real variants. Heterozygosity for sexuals and asexuals was calculated using only sites variable within the ingroup (instead of using complete ORF alignments including the outgroup, as in Kočí et al. [2020]). Expected heterozygosity for diploid asexual hybrids was estimated by combining genotypes of *C. elongatoides* and either *C. taenia* or *C. tanaitica*, which were split into pseudohaplotypes (without phasing) and subsequent calculating proportion of heterozygous sites.

To identify groups of hybrid individuals that putatively belong to the same clonal lineage (MLL) descending from single hybridization event, we followed Arnaud-Haond et al. (2007). Specifically, we created a pairwise matrix of distances between all hybrid individuals calculated from SNP mismatches. We then investigated a histogram of such pairwise distances and found a saddle point, which putatively defines a threshold distance between pairs of individuals belonging to the same or different clones.

The code and scripts for data transfer between databases and basic calculations including MLL identity has been deposited at: https://github.com/cobitislab/LOH-scripts.

### Selection of Species-Specific SNPs, Detection of LOH Events, and Evaluation of Their Topological Clustering

All SNPs that passed through aforementioned filters were attributed into one of the ten categories according to their distribution among biotypes, following Ament-Velásquez et al. (2016); see supplementary table S2, Supplementary Material online. The most important category of SNPs for this study are the species-specific variants (categories shared sh3a-b), where parental species are fixed (monomorphic) for different alleles, thereby allowing for detection of so-called LOH events, where hybrids appear homozygous contrary to the expectation. Having detected the LOH variants in hybrids, we test whether observed LOH events tend to be randomly distributed across individual’s genes or rather tend to cluster, in which case the SNPs with LOH in given gene occur in tight proximity to each other with no discontinuation by heterozygous diagnostic sites. To do so, we identified within each gene *g* the uninterrupted stretches of diagnostic sites with LOH and assigned each such cluster *i* with a score (*S*) so that if the length of the LOH cluster *i* = *n_i_*, then *S_i_ = n_i_^*2. Overall clustering score per animal is then simply represented by *∑_g_∑_i_S*_*i*_ (note that raising to the power of 2 puts higher weight to uninterrupted clusters of LOH events, so that genes with *n* clustered LOH sites would obtain higher score *S* than genes with equal amount of *n* LOH sites that are not contiguous). To test whether observed clustering is nonrandom, we permuted for each hybrid individual its LOH sites across all diagnostic sites and genes, calculated *S*, and compared simulated values with empirical scores.

### Analysis of Sequencing Coverage

We calculated the normalized coverage at each LOH site of every individual using the “total read count” approach (Dillies et al. 2013) and estimated the so-called “relative coverage” by dividing hybrid’s normalized coverage at given site by normalized coverages of the same site in parental species. Since deletions are unlikely in sexual species, this allows detection of hemizygous deletions in hybrids. The expected values then depend on the ploidy of given hybrid: Approximately 1 would indicate the same number of allelic copies indicating a conversion event, whereas hemizygous deletions would generate relative values approximately 0.5 and approximately 0.66 in diploid and triploid hybrids, respectively, whereas double allelic deletion in triploids would generate values approximately 0.33.

To reveal whether observed LOH events in hybrids are generated by gene conversion or hemizygous deletions, or their combination, we performed two tests. First, constructed histograms of relative coverages of all detected LOH sites for each hybrid biotype and tested their modality at values biologically relevant for gene conversion (∼1) or deletion (∼0.5 in diploids, or 0.66 and 0.33 in triploids) (Tucker et al. 2013). To test whether observed distributions deviate from unimodality and to evaluate the relative contribution of gene conversion and deletion processes, we applied the nonlinear least square method implemented in Gnuplot software to consecutively fit each histogram by gamma distributions with the shape parameter *k* optimized by the fitting algorithm and mean (*μ = α/β*) fixed at aforementioned relevant values. In case of diploids, we fitted two distributions (or their mix), centered at 1 and 0.5, whereas in triploids we fitted three distributions (or their mixes) centered at 1, 0.66, and 0.33. Before fittings, we followed the Freedman–Diaconis rule to select the width of the bins in each histogram (Freedman and Diaconis 1981) in order to take into account the properties of particular data sets of each biotype. In case of mix of distributions, we let the fitting algorithm estimate the optimal values of A, B, and C parameters describing relative contributions of individual distributions.

We then simulated how the distribution of normalized coverages should look like, where it generated by conversions only. To do so, we generated simulated data set for each hybrid individual, where coverage values at its LOH sites were sampled from exactly the same number of sites/genes in parental individuals. As such, we obtained realistic null expectation of relative coverages while taking into account used methodology of DNA sequencing and bioinformatic treatment. Such a null distribution simulated for each hybrid biotype has been compared with the empirical one by Kolmogorov–Smirnov test.

### Testing the Effect of Gene/Allele Expressions on LOH Occurrence

To evaluate potential effects of gene/allelic expression on the occurrence of LOH, we compared the present data with those published by Bartoš et al. (2019), who investigated gene expression in parental species *C. elongatoides* and *C. taenia* and in their diploid (ET) and triploid (EET, ETT) hybrids.

Using present gDNA data, we categorized loci based on the presence or the absence of LOH (LOH positive or negative) and its direction (E-like or T-like). We then analyzed the RNA expression of corresponding loci in data of Bartoš et al. (2019) using two tests:

- *Differences in overall gene expression*: To compare the expression levels of LOH-positive and LOH-negative genes in each hybrid biotype, we normalized original read counts by TPM method (transcripts per kilobase million) instead of DeSeq2 method used by Bartoš et al. (2019), since the TPM allowed for comparisons of multiple loci within each individual. The TPM normalization was performed according to Mortazavi et al. (2008).
- *Allelic expression*: Using allele-specific expression data from Bartoš et al. (2019), we also investigated expression divergences between hybrids’ subgenomes in relation to LOH events discovered in this study. Specifically, we used allele-specific data normalized by the total-count approach and tested whether the direction of LOH event (either E-like or T-like) is related to log2 fold change between hybrid’s alleles (Ehyb/Thyb log2FC). The test was performed by comparing the distributions of log2FC values in LOH-negative genes with those of either E-like LOH or T-like LOH-positive genes using WMW test corrected by FDR method for multiple testing. We considered the corrected *P*-value <0.05 as significant.

### Analysis of GO Term Enrichment

The reference transcriptome was annotated with BLAST2GO tool v1.4.4 using GO database as of July 2019). From 20,600 sequences, a subset of 13,557 received BLASTx hit (*e*-value < 0.0001), from which 11,314 was associated with significant GO term annotation (default BLAST2GO settings). To identify GO terms potentially associated with LOH-positive genes, we performed GO enrichment analysis restricted to those genes, which possessed diagnostic sites, thereby technically allowing detection of LOH. *P*-values were calculated from hypergeometric distribution implemented in GO::TermFinder (Boyle et al. 2004) using the list of LOH-positive genes as a testing data set for each biotype. Since gene ontologies terms are a part of acyclic directed graphs (parent and child terms are not independent), we also corrected obtained *P-*values with permutation-based correction provided in GO::TermFinder.

## Supplementary Material

Supplementary data are available at *Molecular Biology and Evolution* online.

## Supplementary Material

msab249_Supplementary_Data
