# Supplementary material for: Genome Fractionation and Loss of Heterozygosity in Hybrids and Polyploids: Mechanisms, Consequences for Selection, and Link to Gene Function
Source: Mol Biol Evol. 2021 Aug 19;38(12):5255–74. doi: 10.1093/molbev/msab249 (PMC8662595; doi:10.1093/molbev/msab249)
Supplement: msab249_Supplementary_Data [file msab249_supplementary_data.zip › appendix_S1_revfin.docx]

Appendix S1

To evaluate whether sequence capture data are suited to compare coverages among loci and across individuals, we investigated the variance in sequencing depth by counting in each sample the number of mapped reads per contig using Bedtools multicov v2.25.0, with default settings (Quinlan and Hall 2010). Subsequently, we built correlation matrix of non-normalized per-contig coverages between all specimens to test if among-locus variances in coverage are similar across individuals. Results for representative sample pairs are demonstrated on Figure 1 and correlation matrix among all analyzed samples is demonstrated on Figure 2.


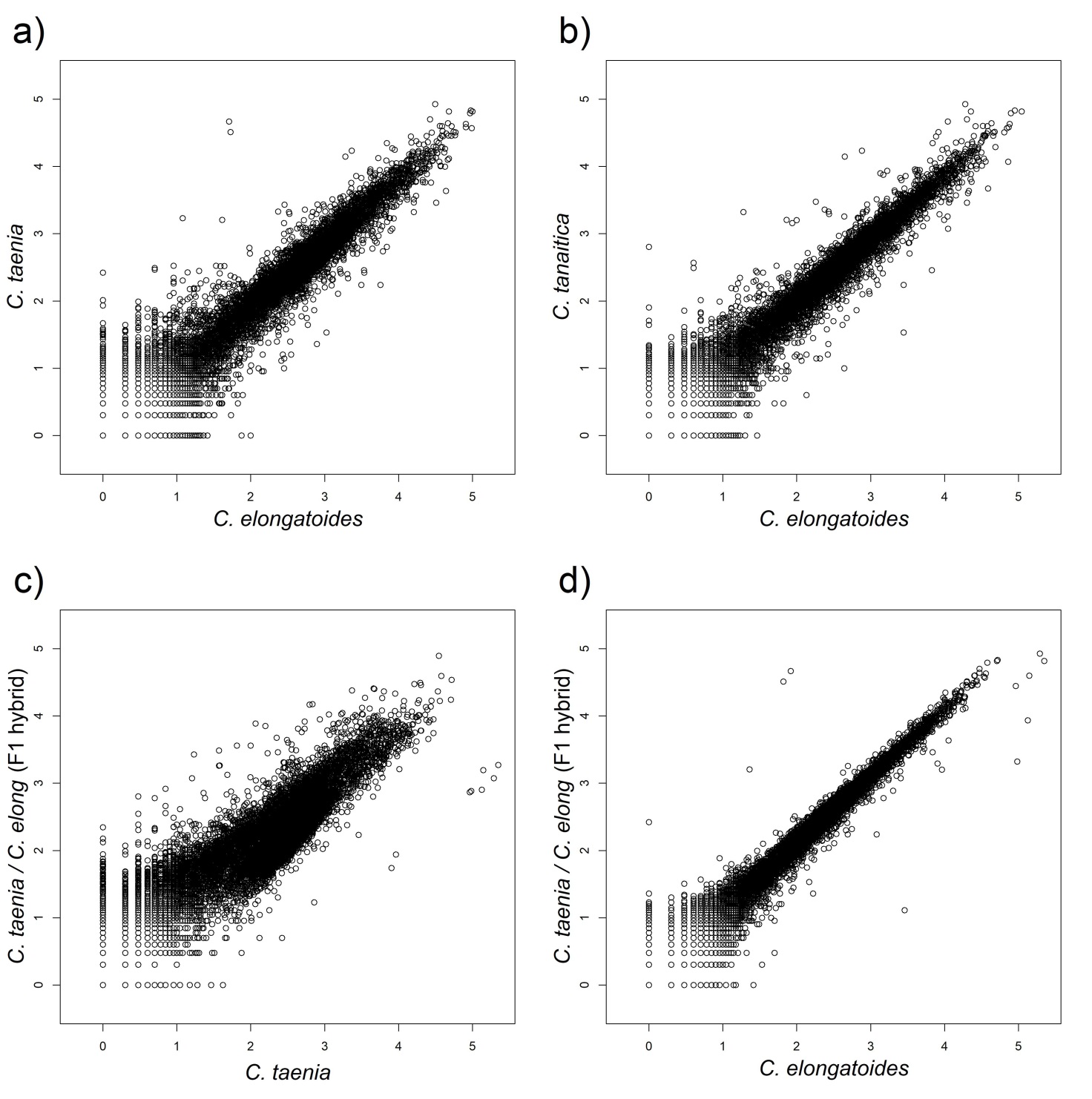


**Figure 1:** Scatter plots indicate correlation of per-contig coverages between randomly chosen representatives of *C. taenia* versus *C. elongatoides* (panel a; Pearson’s R^2^ = 0.9302329) *C. tanaitica* vs *C. elongatoides* (panel b; R^2^ = 0.9383583) and between F1 hybrid sample csc069 and *C. taenia* (panel c; R^2^ = 0.8399942) and and *C. elongatoides* (panel d; R^2^ = 0.9524093). Each point refers to single contig and values along axes indicate log_10_ number of mapped reads per sample and contig; 1 is added to each value to visualize contigs with no coverage.


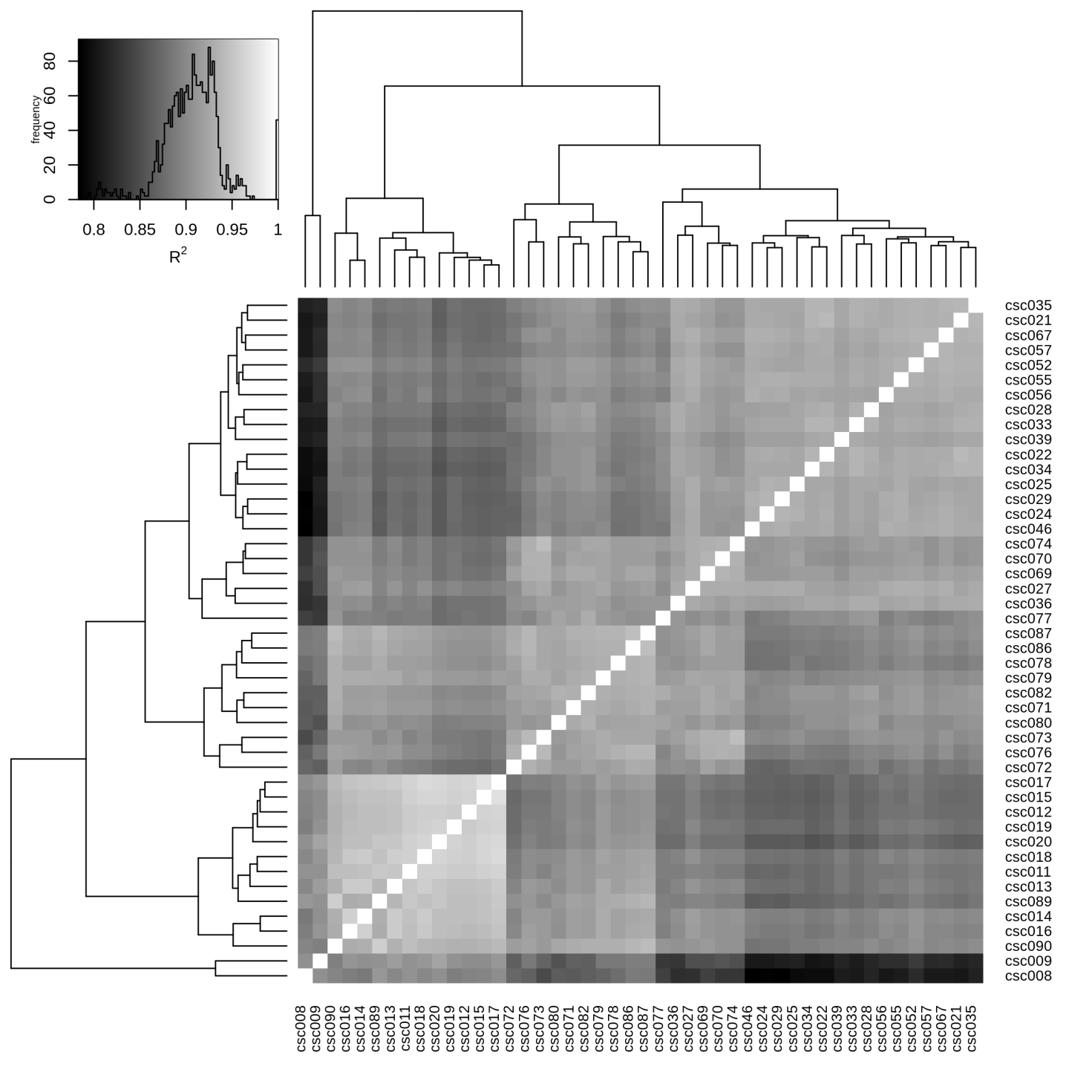

**Figure 2:** Correlation matrix visualized as heatmap: coverage per contig between 46 utilized samples; coverage is not normalized; samples are clustered based on Pearson's correlation coefficient by Euclidean distance.

## References

Quinlan AR, Hall IM. 2010. BEDTools: a flexible suite of utilities for comparing genomic features. Bioinforma. Oxf. Engl. 26:841–842.
