# Supplementary material for: Genome Fractionation and Loss of Heterozygosity in Hybrids and Polyploids: Mechanisms, Consequences for Selection, and Link to Gene Function
Source: Mol Biol Evol. 2021 Aug 19;38(12):5255–74. doi: 10.1093/molbev/msab249 (PMC8662595; doi:10.1093/molbev/msab249)
Supplement: msab249_Supplementary_Data [file msab249_supplementary_data.zip › Figure_S1_revfin.docx]

Figure S1


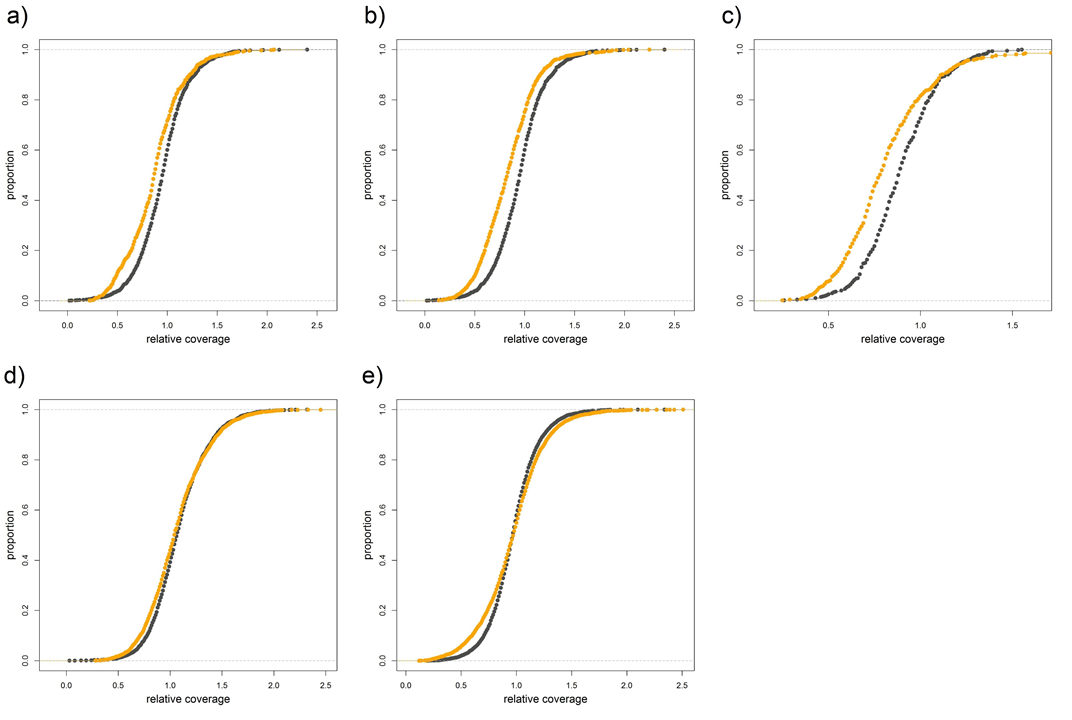


**Figure S1:** Empirical cumulative distribution functions (ECDF) for pools of individual belonging to respective biotypes. Orange represents empirical cumulative distribution function (ECDF) of relative coverages at LOH sites of each hybrid biotype, black represents ECDF of relative coverages at the same sites, but taken from parental species, where no deletions are expected. Significance of differences between curves was tested by Kolmogorov-Smirnov test (KS). Hybrid biotypes: **a)** ET biotype (KS p.val= 8.096e-10), **b)** EET biotype (KS p.val= 2.2e-16), **c)** ETT biotype (KS p.val= 5.323e-12), **d)** EN biotype (KS p.val= 5.395e-6), **e)** EEN biotype (KS p.val= 9.326e-15).
